# Supplementary material for: Computational design of a broad-spectrum multi-epitope vaccine candidate against seven strains of human coronaviruses
Source: 3 Biotech. 2022 Aug 23;12(9):240. doi: 10.1007/s13205-022-03286-0 (PMC9395775; doi:10.1007/s13205-022-03286-0)
Supplement: Supplementary file 1 — Supplementary file1 (PDF 1175 KB) [file 13205_2022_3286_MOESM1_ESM.pdf]

Supplementary Information

Computational design of a broad-spectrum multi-epitope vaccine candidate against seven strains of human coronaviruses

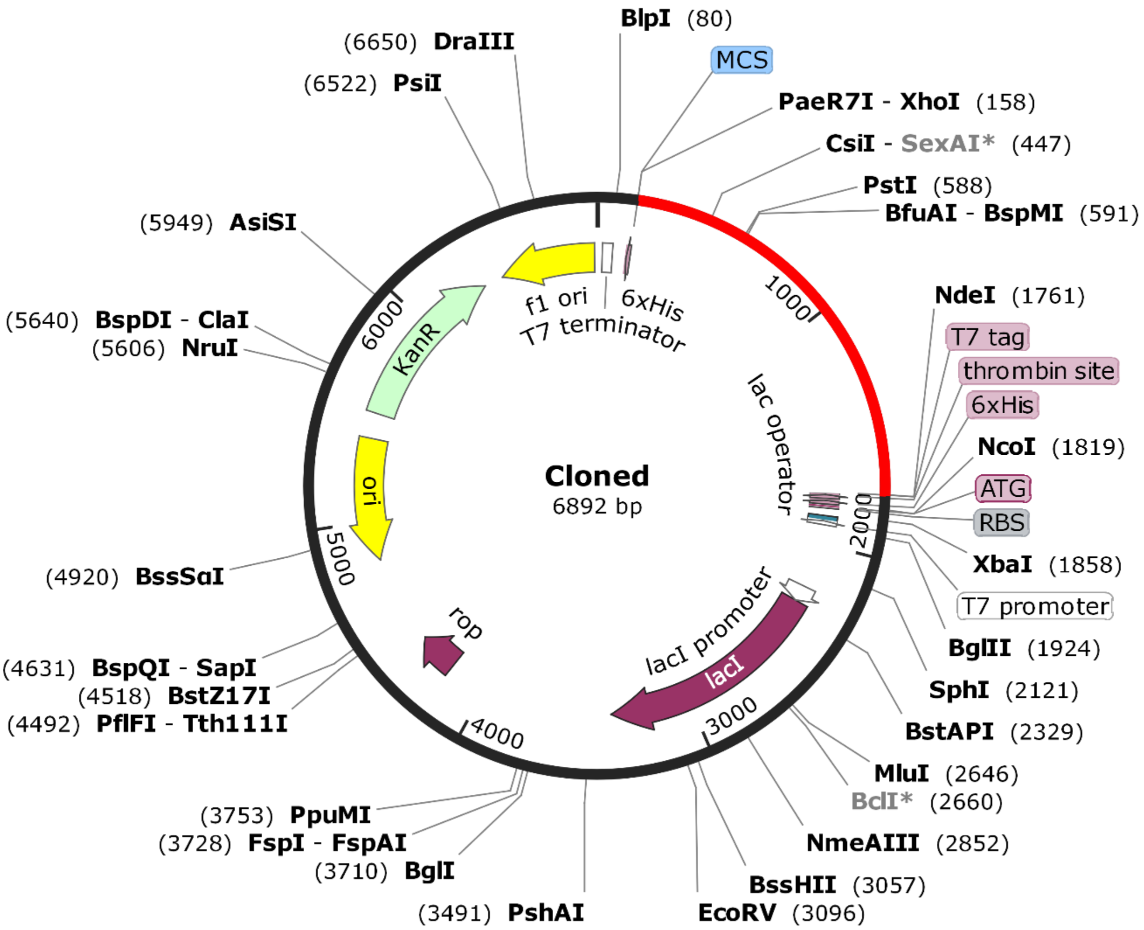

Fig. S1 *In silico* cloning of the final multi-epitope vaccine candidate into the pET28a (+) expression vector where the red portion depicts the gene coding region for the vaccine and the black circle shows the vector backbone.

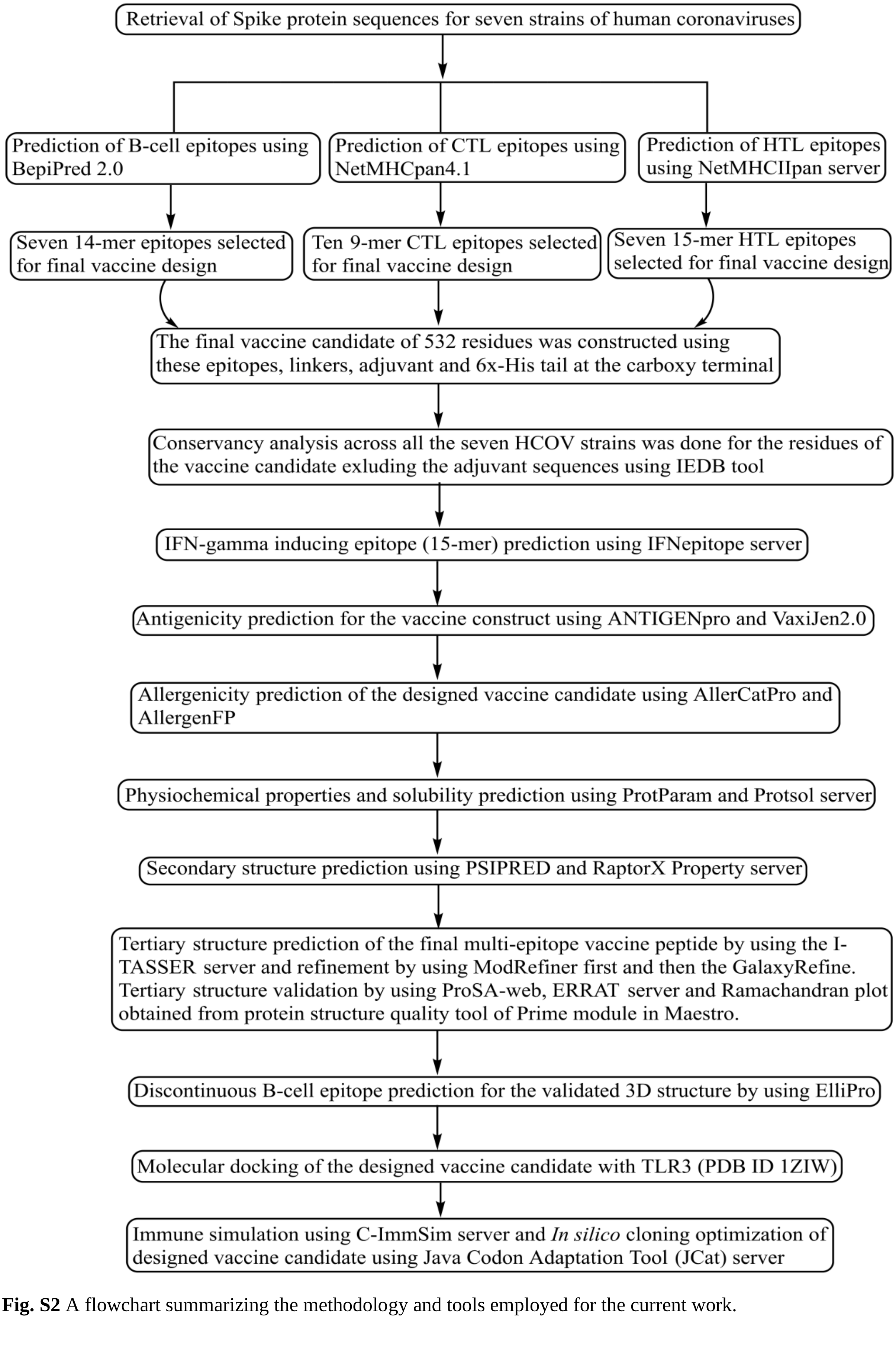

Fig. S2 A flowchart summarizing the methodology and tools employed for the current work.

Table S1 Top 50 CTL epitopes predicted by IEDB for the strain HCoV-229E.

allele	seq_num	start	end	length	peptide	core	icore	score	rank
HLA-A*24:02	1	201	209	9	FYINGYRYF	FYINGYRYF	FYINGYRYF	0.989425	0.01
HLA-A*24:02	1	46	54	9	GYIPSNFAF	GYIPSNFAF	GYIPSNFAF	0.978154	0.01
HLA-A*24:02	1	726	734	9	QYYNGIMVL	QYYNGIMVL	QYYNGIMVL	0.927574	0.04
HLA-A*24:02	1	1057	1065	9	QYNQTILNL	QYNQTILNL	QYNQTILNL	0.893078	0.07
HLA-A*24:02	1	89	97	9	QFTTGFVYF	QFTTGFVYF	QFTTGFVYF	0.888473	0.07
HLA-A*24:02	1	235	243	9	SYADVLVNV	SYADVLVNV	SYADVLVNV	0.862224	0.08
HLA-A*24:02	1	1030	1038	9	EYIDVNKTL	EYIDVNKTL	EYIDVNKTL	0.826523	0.1
HLA-A*24:02	1	299	307	9	TFIVLHVNF	TFIVLHVNF	TFIVLHVNF	0.816799	0.1
HLA-A*24:02	1	986	994	9	NYYRITSRI	NYYRITSRI	NYYRITSRI	0.816488	0.1
HLA-A*24:02	1	1042	1050	9	SYKLPNYTV	SYKLPNYTV	SYKLPNYTV	0.810134	0.11
HLA-A*24:02	1	294	302	9	VYHKHTFIV	VYHKHTFIV	VYHKHTFIV	0.808213	0.11
HLA-A*24:02	1	1162	1170	9	YYDVEKIHI	YYDVEKIHI	YYDVEKIHI	0.77125	0.13
HLA-A*24:02	1	82	90	9	LWSVSGSQF	LWSVSGSQF	LWSVSGSQF	0.756498	0.14
HLA-A*24:02	1	1108	1116	9	TYIKWPWWV	TYIKWPWWV	TYIKWPWWV	0.73653	0.16
HLA-A*24:02	1	995	1003	9	MFEPRIPTI	MFEPRIPTI	MFEPRIPTI	0.709073	0.17
HLA-A*24:02	1	651	659	9	TFDKKAFTL	TFDKKAFTL	TFDKKAFTL	0.665087	0.22
HLA-A*24:02	1	185	193	9	ALPKTVREF	ALPKTVREF	ALPKTVREF	0.659894	0.23
HLA-A*24:02	1	166	174	9	FYCFVNTTI	FYCFVNTTI	FYCFVNTTI	0.560353	0.31
HLA-A*24:02	1	568	576	9	SYDSVSAIV	SYDSVSAIV	SYDSVSAIV	0.555713	0.32
HLA-A*24:02	1	51	59	9	NFAFNNWFL	NFAFNNWFL	NFAFNNWFL	0.51906	0.37
HLA-A*24:02	1	971	979	9	GYVLRQPNL	GYVLRQPNL	GYVLRQPNL	0.499024	0.39
HLA-A*24:02	1	795	803	9	SFNKAMTNI	SFNKAMTNI	SFNKAMTNI	0.49064	0.41
HLA-A*24:02	1	1101	1109	9	KWLNRVETY	KWLNRVETY	KWLNRVETY	0.464291	0.44
HLA-A*24:02	1	623	631	9	QYTSACKTI	QYTSACKTI	QYTSACKTI	0.461581	0.44
HLA-A*24:02	1	142	150	9	VFYCTNNTL	VFYCTNNTL	VFYCTNNTL	0.430188	0.48
HLA-A*24:02	1	470	478	9	TYTSTSGNL	TYTSTSGNL	TYTSTSGNL	0.415268	0.5
HLA-A*24:02	1	161	169	9	TVLGNFYCF	TVLGNFYCF	TVLGNFYCF	0.406085	0.52

HLA-A*24:02	1	379	387	9	KFGSVCFSL	KFGSVCFSL	KFGSVCFSL	0.401393	0.52
HLA-A*24:02	1	449	457	9	IYDVSGVGV	IYDVSGVGV	IYDVSGVGV	0.385271	0.54
HLA-A*24:02	1	66	74	9	VVDGVVRSF	VVDGVVRSF	VVDGVVRSF	0.367721	0.57
HLA-A*24:02	1	799	807	9	AMTNIVDAF	AMTNIVDAF	AMTNIVDAF	0.358389	0.59
HLA-A*24:02	1	200	208	9	HFYINGYRY	HFYINGYRY	HFYINGYRY	0.355952	0.6
HLA-A*24:02	1	1125	1133	9	IFVVSMLLL	IFVVSMLLL	IFVVSMLLL	0.326434	0.67
HLA-A*24:02	1	657	665	9	FTLANVSSF	FTLANVSSF	FTLANVSSF	0.326347	0.67
HLA-A*24:02	1	143	151	9	FYCTNNTLV	FYCTNNTLV	FYCTNNTLV	0.313783	0.7
HLA-A*24:02	1	369	377	9	FSFGKVNNF	FSFGKVNNF	FSFGKVNNF	0.310411	0.71
HLA-A*24:02	1	852	860	9	NFQAISSSI	NFQAISSSI	NFQAISSSI	0.299365	0.74
HLA-A*24:02	1	538	546	9	TYNCTDAVL	TYNCTDAVL	TYNCTDAVL	0.290093	0.77
HLA-A*24:02	1	1047	1055	9	NYTVPDLVV	NYTVPDLVV	NYTVPDLVV	0.252158	0.87
HLA-A*24:02	1	987	995	9	YYRITSRIM	YYRITSRIM	YYRITSRIM	0.230528	0.93
HLA-A*24:02	1	193	201	9	FVISRTGHF	FVISRTGHF	FVISRTGHF	0.225757	0.96
HLA-A*24:02	1	1116	1124	9	VWLCISVVL	VWLCISVVL	VWLCISVVL	0.21416	1
HLA-A*24:02	1	323	331	9	VINITLANF	VINITLANF	VINITLANF	0.214077	1
HLA-A*24:02	1	531	539	9	FFYASNGTY	FFYASNGTY	FFYASNGTY	0.212305	1.1
HLA-A*24:02	1	473	481	9	STSGNLLGF	STSGNLLGF	STSGNLLGF	0.210486	1.1
HLA-A*24:02	1	370	378	9	SFGKVNNFV	SFGKVNNFV	SFGKVNNFV	0.192465	1.1
HLA-A*24:02	1	52	60	9	FAFNNWFLL	FAFNNWFLL	FAFNNWFLL	0.175189	1.2
HLA-A*24:02	1	806	814	9	AFTGVNDAI	AFTGVNDAI	AFTGVNDAI	0.165884	1.3
HLA-A*24:02	1	937	945	9	NAAPEGLVF	NAAPEGLVF	NAAPEGLVF	0.162311	1.3

Table S2 Top 50 CTL epitopes predicted by IEDB for the strain HCoV-HKU1.

allele	seq_num	start	end	length	peptide	core	icore	score	rank
HLA-A*24:02	1	55	63	9	VYLNTTLLF	VYLNTTLLF	VYLNTTLLF	0.993397	0.01
HLA-A*24:02	1	706	714	9	SFISQPFYF	SFISQPFYF	SFISQPFYF	0.988175	0.01
HLA-A*24:02	1	699	707	9	SYVLNNISF	SYVLNNISF	SYVLNNISF	0.971697	0.01
HLA-A*24:02	1	66	74	9	YFPKSGANF	YFPKSGANF	YFPKSGANF	0.967483	0.02
HLA-A*24:02	1	640	648	9	AYYNNWQNL	AYYNNWQNL	AYYNNWQNL	0.957042	0.02

HLA-A*24:02	1	641	649	9	YYNNWQNLL	YYNNWQNLL	YYNNWQNLL	0.946326	0.03
HLA-A*24:02	1	767	775	9	TFEPFNVSF	TFEPFNVSF	TFEPFNVSF	0.93064	0.03
HLA-A*24:02	1	1291	1299	9	MYVKWPWYI	MYVKWPWYI	MYVKWPWYI	0.90732	0.06
HLA-A*24:02	1	1139	1147	9	PYGLLFIHF	PYGLLFIHF	PYGLLFIHF	0.894727	0.07
HLA-A*24:02	1	27	35	9	HYNKTIPRI	HYNKTIPRI	HYNKTIPRI	0.89014	0.07
HLA-A*24:02	1	129	137	9	VFVNTSYTI	VFVNTSYTI	VFVNTSYTI	0.874186	0.07
HLA-A*24:02	1	1294	1302	9	KWPWYIWLL	KWPWYIWLL	KWPWYIWLL	0.852592	0.08
HLA-A*24:02	1	49	57	9	YYVLNRVYL	YYVLNRVYL	YYVLNRVYL	0.84829	0.09
HLA-A*24:02	1	1177	1185	9	YFIKQNDSW	YFIKQNDSW	YFIKQNDSW	0.784331	0.12
HLA-A*24:02	1	1299	1307	9	IWLLIVILF	IWLLIVILF	IWLLIVILF	0.783527	0.13
HLA-A*24:02	1	303	311	9	VYDLSGFTV	VYDLSGFTV	VYDLSGFTV	0.782314	0.13
HLA-A*24:02	1	216	224	9	VGMPTTFLF	VGMPTTFLF	VGMPTTFLF	0.772628	0.13
HLA-A*24:02	1	447	455	9	RYGFGSFNV	RYGFGSFNV	RYGFGSFNV	0.755253	0.14
HLA-A*24:02	1	1146	1154	9	HFSYKPTSF	HFSYKPTSF	HFSYKPTSF	0.720147	0.17
HLA-A*24:02	1	426	434	9	YYSLPLLNV	YYSLPLLNV	YYSLPLLNV	0.684208	0.2
HLA-A*24:02	1	1237	1245	9	LWFKNHTSI	LWFKNHTSI	LWFKNHTSI	0.672232	0.21
HLA-A*24:02	1	1258	1266	9	TFLDLYYEM	TFLDLYYEM	TFLDLYYEM	0.671194	0.21
HLA-A*24:02	1	202	210	9	FYQERGVFY	FYQERGVFY	FYQERGVFY	0.657803	0.23
HLA-A*24:02	1	649	657	9	LYDSNGNII	LYDSNGNII	LYDSNGNII	0.636976	0.25
HLA-A*24:02	1	461	469	9	VYSDHCFSV	VYSDHCFSV	VYSDHCFSV	0.628219	0.25
HLA-A*24:02	1	760	768	9	SSPYRFVTF	SSPYRFVTF	SSPYRFVTF	0.625266	0.26
HLA-A*24:02	1	625	633	9	LYGITGQGI	LYGITGQGI	LYGITGQGI	0.590299	0.29
HLA-A*24:02	1	822	830	9	NYAACHDLL	NYAACHDLL	NYAACHDLL	0.546239	0.33
HLA-A*24:02	1	201	209	9	HFYQERGVF	HFYQERGVF	HFYQERGVF	0.533629	0.35
HLA-A*24:02	1	1008	1016	9	AFNKALLSI	AFNKALLSI	AFNKALLSI	0.48659	0.42
HLA-A*24:02	1	117	125	9	LYSEFSTIV	LYSEFSTIV	LYSEFSTIV	0.480866	0.42
HLA-A*24:02	1	190	198	9	TYNVSADWL	TYNVSADWL	TYNVSADWL	0.448943	0.46
HLA-A*24:02	1	795	803	9	FTIAGHEEF	FTIAGHEEF	FTIAGHEEF	0.429553	0.48
HLA-A*24:02	1	757	765	9	RGISSPYRF	RGISSPYRF	RGISSPYRF	0.423999	0.49

HLA-A*24:02	1	1051	1059	9	KFGAISSSL	KFGAISSSL	KFGAISSSL	0.423223	0.49
HLA-A*24:02	1	835	843	9	TFCDNINSI	TFCDNINSI	TFCDNINSI	0.423197	0.49
HLA-A*24:02	1	1230	1238	9	DFEAEFSLW	DFEAEFSLW	DFEAEFSLW	0.417342	0.5
HLA-A*24:02	1	789	797	9	IQIPTNFTI	IQIPTNFTI	IQIPTNFTI	0.407913	0.51
HLA-A*24:02	1	1217	1225	9	PFIYLNNSI	PFIYLNNSI	PFIYLNNSI	0.404024	0.52
HLA-A*24:02	1	1243	1251	9	TSIAPNLTF	TSIAPNLTF	TSIAPNLTF	0.386378	0.54
HLA-A*24:02	1	48	56	9	TYYVLNRVY	TYYVLNRVY	TYYVLNRVY	0.362417	0.57
HLA-A*24:02	1	1044	1052	9	LLQQLFNKF	LLQQLFNKF	LLQQLFNKF	0.346703	0.62
HLA-A*24:02	1	348	356	9	IFSNCNFNL	IFSNCNFNL	IFSNCNFNL	0.338473	0.63
HLA-A*24:02	1	1136	1144	9	QNAPYGLLF	QNAPYGLLF	QNAPYGLLF	0.311839	0.71
HLA-A*24:02	1	378	386	9	IFGSCFNSI	IFGSCFNSI	IFGSCFNSI	0.297154	0.75
HLA-A*24:02	1	296	304	9	SFAPNTGVY	SFAPNTGVY	SFAPNTGVY	0.289835	0.77
HLA-A*24:02	1	192	200	9	NVSADWLYF	NVSADWLYF	NVSADWLYF	0.247951	0.88
HLA-A*24:02	1	633	641	9	IFKEVSAAY	IFKEVSAAY	IFKEVSAAY	0.242186	0.89
HLA-A*24:02	1	1184	1192	9	SWMFTGSSY	SWMFTGSSY	SWMFTGSSY	0.236748	0.91

Table S3 Top 50 CTL epitopes predicted by IEDB for the strain HCoV-NL63.

allele	seq_num	start	end	length	peptide	core	icore	score	rank
HLA-A*24:02	1	384	392	9	FYINGFKYF	FYINGFKYF	FYINGFKYF	0.988632	0.01
HLA-A*24:02	1	479	487	9	YYQHTDINF	YYQHTDINF	YYQHTDINF	0.982551	0.01
HLA-A*24:02	1	160	168	9	FYVPAAYKL	FYVPAAYKL	FYVPAAYKL	0.976439	0.01
HLA-A*24:02	1	87	95	9	YYIYVTNEI	YYIYVTNEI	YYIYVTNEI	0.953534	0.03
HLA-A*24:02	1	909	917	9	QYYNGIMVL	QYYNGIMVL	QYYNGIMVL	0.927574	0.04
HLA-A*24:02	1	81	89	9	YYDVNQYYI	YYDVNQYYI	YYDVNQYYI	0.914726	0.06
HLA-A*24:02	1	412	420	9	FWTVAFATF	FWTVAFATF	FWTVAFATF	0.859301	0.08
HLA-A*24:02	1	1299	1307	9	VWLIISVVF	VWLIISVVF	VWLIISVVF	0.845854	0.09
HLA-A*24:02	1	1244	1252	9	TYLNLSSEL	TYLNLSSEL	TYLNLSSEL	0.809381	0.11
HLA-A*24:02	1	632	640	9	IYDYVGTGI	IYDYVGTGI	IYDYVGTGI	0.79311	0.12
HLA-A*24:02	1	1230	1238	9	KYVKPNFDL	KYVKPNFDL	KYVKPNFDL	0.779867	0.13
HLA-A*24:02	1	349	357	9	YYCFINSTI	YYCFINSTI	YYCFINSTI	0.747461	0.15

HLA-A*24:02	1	212	220	9	GYTDNIFSV	GYTDNIFSV	GYTDNIFSV	0.7286	0.16
HLA-A*24:02	1	57	65	9	VYSANGFFY	VYSANGFFY	VYSANGFFY	0.726991	0.17
HLA-A*24:02	1	584	592	9	TWHYTSYTI	TWHYTSYTI	TWHYTSYTI	0.720905	0.17
HLA-A*24:02	1	89	97	9	IYVTNEIGL	IYVTNEIGL	IYVTNEIGL	0.702214	0.18
HLA-A*24:02	1	80	88	9	GYYDVNQYY	GYYDVNQYY	GYYDVNQYY	0.689936	0.19
HLA-A*24:02	1	1178	1186	9	MFQPRLPVL	MFQPRLPVL	MFQPRLPVL	0.676538	0.21
HLA-A*24:02	1	1294	1302	9	KWPWWVWLI	KWPWWVWLI	KWPWWVWLI	0.673354	0.21
HLA-A*24:02	1	229	237	9	GFPFNNWFL	GFPFNNWFL	GFPFNNWFL	0.662835	0.22
HLA-A*24:02	1	1213	1221	9	DYVDVNKTL	DYVDVNKTL	DYVDVNKTL	0.659567	0.23
HLA-A*24:02	1	978	986	9	SFNKAINNI	SFNKAINNI	SFNKAINNI	0.647303	0.24
HLA-A*24:02	1	1291	1299	9	NYIKWPWWV	NYIKWPWWV	NYIKWPWWV	0.642158	0.24
HLA-A*24:02	1	472	480	9	TYVALPIYY	TYVALPIYY	TYVALPIYY	0.628136	0.25
HLA-A*24:02	1	653	661	9	TYVSNSGNL	TYVSNSGNL	TYVSNSGNL	0.614775	0.27
HLA-A*24:02	1	589	597	9	SYTIVGALY	SYTIVGALY	SYTIVGALY	0.556293	0.32
HLA-A*24:02	1	834	842	9	TFDSNAFSL	TFDSNAFSL	TFDSNAFSL	0.530278	0.35
HLA-A*24:02	1	1154	1162	9	GYVLRQPNL	GYVLRQPNL	GYVLRQPNL	0.499024	0.39
HLA-A*24:02	1	152	160	9	HLYNVTRTF	HLYNVTRTF	HLYNVTRTF	0.491247	0.41
HLA-A*24:02	1	806	814	9	QYTSACKTI	QYTSACKTI	QYTSACKTI	0.461581	0.44
HLA-A*24:02	1	1035	1043	9	NFQAISNSI	NFQAISNSI	NFQAISNSI	0.441886	0.46
HLA-A*24:02	1	388	396	9	GFKYFDLGF	GFKYFDLGF	GFKYFDLGF	0.437955	0.47
HLA-A*24:02	1	1347	1355	9	YYEFEKVHV	YYEFEKVHV	YYEFEKVHV	0.424865	0.49
HLA-A*24:02	1	1240	1248	9	PFNLTYLNL	PFNLTYLNL	PFNLTYLNL	0.42405	0.49
HLA-A*24:02	1	468	476	9	VLPETYVAL	VLPETYVAL	VLPETYVAL	0.41577	0.5
HLA-A*24:02	1	9	17	9	VLPLASCFF	VLPLASCFF	VLPLASCFF	0.411851	0.51
HLA-A*24:02	1	175	183	9	CYFNYSCVF	CYFNYSCVF	CYFNYSCVF	0.396797	0.53
HLA-A*24:02	1	1235	1243	9	NFDLTPFNL	NFDLTPFNL	NFDLTPFNL	0.387673	0.54
HLA-A*24:02	1	368	376	9	ILPPTVREI	ILPPTVREI	ILPPTVREI	0.385522	0.54
HLA-A*24:02	1	416	424	9	AFATFVDVL	AFATFVDVL	AFATFVDVL	0.372098	0.57
HLA-A*24:02	1	1342	1350	9	STKLPYYEF	STKLPYYEF	STKLPYYEF	0.364429	0.57

HLA-A*24:02	1	1192	1200	9	IYNCNVTFV	IYNCNVTFV	IYNCNVTFV	0.352104	0.61
HLA-A*24:02	1	267	275	9	KSSTGFVYF	KSSTGFVYF	KSSTGFVYF	0.342751	0.62
HLA-A*24:02	1	318	326	9	KTLQYDVLF	KTLQYDVLF	KTLQYDVLF	0.331881	0.65
HLA-A*24:02	1	182	190	9	VFSVVNATV	VFSVVNATV	VFSVVNATV	0.323667	0.67
HLA-A*24:02	1	123	131	9	SFDCIVNLL	SFDCIVNLL	SFDCIVNLL	0.319792	0.69
HLA-A*24:02	1	592	600	9	IVGALYVTW	IVGALYVTW	IVGALYVTW	0.302827	0.73
HLA-A*24:02	1	982	990	9	AINNIVASF	AINNIVASF	AINNIVASF	0.299454	0.74
HLA-A*24:02	1	840	848	9	FSLANVTSF	FSLANVTSF	FSLANVTSF	0.297235	0.75

Table S4 Top 50 CTL epitopes predicted by IEDB for the strain HCoV-OC43.

allele	seq_num	start	end	length	peptide	core	icore	score	rank
HLA-A*24:02	1	645	653	9	TYYNSWQNL	TYYNSWQNL	TYYNSWQNL	0.960391	0.02
HLA-A*24:02	1	1184	1192	9	YFVNVNNTW	YFVNVNNTW	YFVNVNNTW	0.94727	0.03
HLA-A*24:02	1	61	69	9	VYLNTTLFL	VYLNTTLFL	VYLNTTLFL	0.94248	0.03
HLA-A*24:02	1	646	654	9	YYNSWQNLL	YYNSWQNLL	YYNSWQNLL	0.941893	0.03
HLA-A*24:02	1	987	995	9	LYINVQYRI	LYINVQYRI	LYINVQYRI	0.937508	0.03
HLA-A*24:02	1	1146	1154	9	PYGLYFIHF	PYGLYFIHF	PYGLYFIHF	0.907312	0.06
HLA-A*24:02	1	320	328	9	VYELNGYTV	VYELNGYTV	VYELNGYTV	0.853988	0.08
HLA-A*24:02	1	1297	1305	9	YYVKWPWYV	YYVKWPWYV	YYVKWPWYV	0.846746	0.09
HLA-A*24:02	1	1300	1308	9	KWPWYVWLL	KWPWYVWLL	KWPWYVWLL	0.844141	0.09
HLA-A*24:02	1	55	63	9	YYVLDRVYL	YYVLDRVYL	YYVLDRVYL	0.842726	0.09
HLA-A*24:02	1	220	228	9	HFYQEGGTF	HFYQEGGTF	HFYQEGGTF	0.802938	0.11
HLA-A*24:02	1	1219	1227	9	NYTKAPYVM	NYTKAPYVM	NYTKAPYVM	0.795037	0.12
HLA-A*24:02	1	286	294	9	AFNQDGIIF	AFNQDGIIF	AFNQDGIIF	0.791547	0.12
HLA-A*24:02	1	209	217	9	TYDVNATYL	TYDVNATYL	TYDVNATYL	0.754379	0.14
HLA-A*24:02	1	714	722	9	QLQPINYSF	QLQPINYSF	QLQPINYSF	0.741558	0.16
HLA-A*24:02	1	1224	1232	9	PYVMLNTSI	PYVMLNTSI	PYVMLNTSI	0.73866	0.16
HLA-A*24:02	1	443	451	9	YYNLPAANV	YYNLPAANV	YYNLPAANV	0.72402	0.17
HLA-A*24:02	1	72	80	9	YYPTSGSTY	YYPTSGSTY	YYPTSGSTY	0.713792	0.17
HLA-A*24:02	1	221	229	9	FYQEGGTFY	FYQEGGTFY	FYQEGGTFY	0.673462	0.21

HLA-A*24:02	1	60	68	9	RVYLNTTLF	RVYLNTTLF	RVYLNTTLF	0.660858	0.23
HLA-A*24:02	1	254	262	9	YYVMPLTCI	YYVMPLTCI	YYVMPLTCI	0.599617	0.28
HLA-A*24:02	1	1058	1066	9	RFGAISASL	RFGAISASL	RFGAISASL	0.574888	0.3
HLA-A*24:02	1	732	740	9	AYNSTAISV	AYNSTAISV	AYNSTAISV	0.569683	0.31
HLA-A*24:02	1	883	891	9	NFNVDDINF	NFNVDDINF	NFNVDDINF	0.549158	0.32
HLA-A*24:02	1	638	646	9	IFVEVNATY	IFVEVNATY	IFVEVNATY	0.546969	0.32
HLA-A*24:02	1	630	638	9	LYGISGQGI	LYGISGQGI	LYGISGQGI	0.540051	0.34
HLA-A*24:02	1	1257	1265	9	SLDYINVTF	SLDYINVTF	SLDYINVTF	0.531368	0.35
HLA-A*24:02	1	1015	1023	9	AFNNALHAI	AFNNALHAI	AFNNALHAI	0.519934	0.37
HLA-A*24:02	1	1193	1201	9	MYTGSGYYY	MYTGSGYYY	MYTGSGYYY	0.505365	0.39
HLA-A*24:02	1	473	481	9	FVPQPTGVF	FVPQPTGVF	FVPQPTGVF	0.484196	0.42
HLA-A*24:02	1	654	662	9	LYDSNGNLY	LYDSNGNLY	LYDSNGNLY	0.465779	0.44
HLA-A*24:02	1	763	771	9	RAITTGYRF	RAITTGYRF	RAITTGYRF	0.454069	0.45
HLA-A*24:02	1	801	809	9	FTIGNMEEF	FTIGNMEEF	FTIGNMEEF	0.443359	0.46
HLA-A*24:02	1	1149	1157	9	LYFIHFNYV	LYFIHFNYV	LYFIHFNYV	0.433638	0.48
HLA-A*24:02	1	828	836	9	DYAACKLQL	DYAACKLQL	DYAACKLQL	0.409523	0.51
HLA-A*24:02	1	395	403	9	IYGMCFSSI	IYGMCFSSI	IYGMCFSSI	0.40747	0.51
HLA-A*24:02	1	666	674	9	DYITNRTFM	DYITNRTFM	DYITNRTFM	0.393099	0.54
HLA-A*24:02	1	841	849	9	SFCDNINAI	SFCDNINAI	SFCDNINAI	0.344295	0.62
HLA-A*24:02	1	230	238	9	AYFTDTGFV	AYFTDTGFV	AYFTDTGFV	0.332705	0.65
HLA-A*24:02	1	1264	1272	9	TFLDLQVEM	TFLDLQVEM	TFLDLQVEM	0.327467	0.67
HLA-A*24:02	1	1244	1252	9	QWFKNQTSV	QWFKNQTSV	QWFKNQTSV	0.322091	0.67
HLA-A*24:02	1	54	62	9	TYYVLDRVY	TYYVLDRVY	TYYVLDRVY	0.305349	0.72
HLA-A*24:02	1	1230	1238	9	TSIPNLPDF	TSIPNLPDF	TSIPNLPDF	0.2899	0.77
HLA-A*24:02	1	838	846	9	EYGSFCDNI	EYGSFCDNI	EYGSFCDNI	0.276575	0.81
HLA-A*24:02	1	135	143	9	TFVNTSYSV	TFVNTSYSV	TFVNTSYSV	0.276323	0.81
HLA-A*24:02	1	8	16	9	SLPTAFAVI	SLPTAFAVI	SLPTAFAVI	0.270781	0.82
HLA-A*24:02	1	472	480	9	VFVPQPTGV	VFVPQPTGV	VFVPQPTGV	0.270527	0.82
HLA-A*24:02	1	795	803	9	IQIPSEFTI	IQIPSEFTI	IQIPSEFTI	0.267764	0.82

HLA-A*24:02	1	1343	1351	9	DYTGYQELV	DYTGYQELV	DYTGYQELV	0.26583	0.83

Table S5 Top 50 CTL epitopes predicted by IEDB for the strain MERS.

allele	seq_num	start	end	length	peptide	core	icore	score	rank
HLA-A*24:02	1	273	281	9	LYGGNMFQF	LYGGNMFQF	LYGGNMFQF	0.989721	0.01
HLA-A*24:02	1	315	323	9	VYKLQPLTF	VYKLQPLTF	VYKLQPLTF	0.981119	0.01
HLA-A*24:02	1	292	300	9	YYSIIPHSI	YYSIIPHSI	YYSIIPHSI	0.980988	0.01
HLA-A*24:02	1	1291	1299	9	YYNKWPWYI	YYNKWPWYI	YYNKWPWYI	0.958668	0.02
HLA-A*24:02	1	1210	1218	9	TYQNISTNL	TYQNISTNL	TYQNISTNL	0.925226	0.04
HLA-A*24:02	1	396	404	9	VYNFKRLVF	VYNFKRLVF	VYNFKRLVF	0.916113	0.05
HLA-A*24:02	1	104	112	9	NYSQDVKQF	NYSQDVKQF	NYSQDVKQF	0.875769	0.07
HLA-A*24:02	1	1141	1149	9	YYPSNHIEV	YYPSNHIEV	YYPSNHIEV	0.8212	0.1
HLA-A*24:02	1	63	71	9	TYSNITITY	TYSNITITY	TYSNITITY	0.805231	0.11
HLA-A*24:02	1	444	452	9	DYFSYPLSM	DYFSYPLSM	DYFSYPLSM	0.788488	0.12
HLA-A*24:02	1	163	171	9	RFFNHTLVL	RFFNHTLVL	RFFNHTLVL	0.783652	0.13
HLA-A*24:02	1	391	399	9	GTPPQVYNF	GTPPQVYNF	GTPPQVYNF	0.699119	0.18
HLA-A*24:02	1	631	639	9	VYDAYQNLV	VYDAYQNLV	VYDAYQNLV	0.685014	0.2
HLA-A*24:02	1	498	506	9	SYINKCSRL	SYINKCSRL	SYINKCSRL	0.682248	0.2
HLA-A*24:02	1	927	935	9	QYVAGYKVL	QYVAGYKVL	QYVAGYKVL	0.677072	0.21
HLA-A*24:02	1	959	967	9	GWTAGLSSF	GWTAGLSSF	GWTAGLSSF	0.668383	0.21
HLA-A*24:02	1	1203	1211	9	KYVAPQVTY	KYVAPQVTY	KYVAPQVTY	0.667127	0.21
HLA-A*24:02	1	231	239	9	YFNLRNCTF	YFNLRNCTF	YFNLRNCTF	0.659277	0.23
HLA-A*24:02	1	1290	1298	9	TYYNKWPWY	TYYNKWPWY	TYYNKWPWY	0.636177	0.25
HLA-A*24:02	1	1183	1191	9	EWSYTGSSF	EWSYTGSSF	EWSYTGSSF	0.568302	0.31
HLA-A*24:02	1	931	939	9	GYKVLPPLM	GYKVLPPLM	GYKVLPPLM	0.548315	0.32
HLA-A*24:02	1	641	649	9	YYSDDGNYY	YYSDDGNYY	YYSDDGNYY	0.526389	0.36
HLA-A*24:02	1	1299	1307	9	IWLGFIAGL	IWLGFIAGL	IWLGFIAGL	0.516336	0.37
HLA-A*24:02	1	1043	1051	9	TFGAISASI	TFGAISASI	TFGAISASI	0.486726	0.42
HLA-A*24:02	1	977	985	9	FYRLNGVGI	FYRLNGVGI	FYRLNGVGI	0.481295	0.42
HLA-A*24:02	1	1011	1019	9	GFTTTNEAF	GFTTTNEAF	GFTTTNEAF	0.470517	0.43

HLA-A*24:02	1	1128	1136	9	VNAPNGLYF	VNAPNGLYF	VNAPNGLYF	0.46258	0.44
HLA-A*24:02	1	242	250	9	TYNITEDEI	TYNITEDEI	TYNITEDEI	0.452318	0.45
HLA-A*24:02	1	317	325	9	KLQPLTFLL	KLQPLTFLL	KLQPLTFLL	0.444888	0.46
HLA-A*24:02	1	780	788	9	LSIPTNFSF	LSIPTNFSF	LSIPTNFSF	0.415181	0.5
HLA-A*24:02	1	258	266	9	QTAQGVHLF	QTAQGVHLF	QTAQGVHLF	0.414327	0.51
HLA-A*24:02	1	1238	1246	9	FFKNVSTSI	FFKNVSTSI	FFKNVSTSI	0.404692	0.52
HLA-A*24:02	1	703	711	9	TYGPLQTPV	TYGPLQTPV	TYGPLQTPV	0.384985	0.54
HLA-A*24:02	1	164	172	9	FFNHTLVLL	FFNHTLVLL	FFNHTLVLL	0.378117	0.55
HLA-A*24:02	1	1230	1238	9	DFQDELDEF	DFQDELDEF	DFQDELDEF	0.365944	0.57
HLA-A*24:02	1	224	232	9	SLNSFKEYF	SLNSFKEYF	SLNSFKEYF	0.362022	0.58
HLA-A*24:02	1	465	473	9	SQFNYKQSF	SQFNYKQSF	SQFNYKQSF	0.347702	0.62
HLA-A*24:02	1	403	411	9	VFTNCNYNL	VFTNCNYNL	VFTNCNYNL	0.335332	0.64
HLA-A*24:02	1	540	548	9	YYRKQLSPL	YYRKQLSPL	YYRKQLSPL	0.327781	0.67
HLA-A*24:02	1	823	831	9	EYGQFCSKI	EYGQFCSKI	EYGQFCSKI	0.321755	0.67
HLA-A*24:02	1	969	977	9	AIPFAQSIF	AIPFAQSIF	AIPFAQSIF	0.308574	0.71
HLA-A*24:02	1	313	321	9	FYVYKLQPL	FYVYKLQPL	FYVYKLQPL	0.306098	0.72
HLA-A*24:02	1	634	642	9	AYQNLVGYY	AYQNLVGYY	AYQNLVGYY	0.303797	0.72
HLA-A*24:02	1	522	530	9	QYSPCVSIV	QYSPCVSIV	QYSPCVSIV	0.28589	0.78
HLA-A*24:02	1	861	869	9	IIPGFGGDF	IIPGFGGDF	IIPGFGGDF	0.285477	0.78
HLA-A*24:02	1	576	584	9	QYGTDTNSV	QYGTDTNSV	QYGTDTNSV	0.279111	0.8
HLA-A*24:02	1	2	10	9	IHSVFLLMF	IHSVFLLMF	IHSVFLLMF	0.272336	0.82
HLA-A*24:02	1	472	480	9	SFSNPTCLI	SFSNPTCLI	SFSNPTCLI	0.268818	0.82
HLA-A*24:02	1	1342	1350	9	EYDLEPHKV	EYDLEPHKV	EYDLEPHKV	0.239935	0.9

Table S6 Top 50 CTL epitopes predicted by IEDB for the strain SARS-CoV.

allele	seq_num	start	end	length	peptide	core	icore	score	rank
HLA-A*24:02	1	607	615	9	VYSTGVNVF	VYSTGVNVF	VYSTGVNVF	0.985578	0.01
HLA-A*24:02	1	461	469	9	VYTLSTYDF	VYTLSTYDF	VYTLSTYDF	0.958617	0.02
HLA-A*24:02	1	262	270	9	KYTTFMLSF	KYTTFMLSF	KYTTFMLSF	0.927854	0.04
HLA-A*24:02	1	1055	1063	9	AYFPREGVF	AYFPREGVF	AYFPREGVF	0.925894	0.04

HLA-A*24:02	1	241	249	9	MFSQFNSNF	MFSQFNSNF	MFSQFNSNF	0.888294	0.07
HLA-A*24:02	1	1176	1184	9	QYIKWPWYV	QYIKWPWYV	QYIKWPWYV	0.881222	0.07
HLA-A*24:02	1	164	172	9	TYDRVEKSF	TYDRVEKSF	TYDRVEKSF	0.872189	0.07
HLA-A*24:02	1	469	477	9	FYPSIPVEY	FYPSIPVEY	FYPSIPVEY	0.872001	0.07
HLA-A*24:02	1	1062	1070	9	VFVSNGTSW	VFVSNGTSW	VFVSNGTSW	0.868845	0.07
HLA-A*24:02	1	234	242	9	SYRVVMTMF	SYRVVMTMF	SYRVVMTMF	0.862988	0.08
HLA-A*24:02	1	81	89	9	YFDNPILDF	YFDNPILDF	YFDNPILDF	0.858904	0.08
HLA-A*24:02	1	1069	1077	9	SWFITQRNF	SWFITQRNF	SWFITQRNF	0.848297	0.09
HLA-A*24:02	1	341	349	9	VYAWERTKI	VYAWERTKI	VYAWERTKI	0.822536	0.1
HLA-A*24:02	1	1105	1113	9	VYDPLQPEL	VYDPLQPEL	VYDPLQPEL	0.81018	0.11
HLA-A*24:02	1	674	682	9	AYANNSIAI	AYANNSIAI	AYANNSIAI	0.798541	0.12
HLA-A*24:02	1	303	311	9	IYQTSNFRV	IYQTSNFRV	IYQTSNFRV	0.784957	0.12
HLA-A*24:02	1	58	66	9	YFLPFDSNL	YFLPFDSNL	YFLPFDSNL	0.64101	0.24
HLA-A*24:02	1	255	263	9	AYYVGNLKY	AYYVGNLKY	AYYVGNLKY	0.569289	0.31
HLA-A*24:02	1	723	731	9	QYGSFCTQL	QYGSFCTQL	QYGSFCTQL	0.561401	0.31
HLA-A*24:02	1	237	245	9	VVMTMFSQF	VVMTMFSQF	VVMTMFSQF	0.549274	0.32
HLA-A*24:02	1	1184	1192	9	VWLGFIAGL	VWLGFIAGL	VWLGFIAGL	0.545431	0.33
HLA-A*24:02	1	212	220	9	GFPAGFSVL	GFPAGFSVL	GFPAGFSVL	0.541095	0.34
HLA-A*24:02	1	46	54	9	IFRSDVLHL	IFRSDVLHL	IFRSDVLHL	0.511313	0.38
HLA-A*24:02	1	336	344	9	SRFPNVYAW	SRFPNVYAW	SRFPNVYAW	0.44071	0.46
HLA-A*24:02	1	894	902	9	QFNKAISQI	QFNKAISQI	QFNKAISQI	0.398213	0.53
HLA-A*24:02	1	848	856	9	GTATAGWTF	GTATAGWTF	GTATAGWTF	0.395854	0.53
HLA-A*24:02	1	1056	1064	9	YFPREGVFV	YFPREGVFV	YFPREGVFV	0.384153	0.54
HLA-A*24:02	1	866	874	9	FAMQMAYRF	FAMQMAYRF	FAMQMAYRF	0.375038	0.56
HLA-A*24:02	1	78	86	9	KYTYFDNPI	KYTYFDNPI	KYTYFDNPI	0.329357	0.66
HLA-A*24:02	1	529	537	9	KRFQSFQQF	KRFQSFQQF	KRFQSFQQF	0.278466	0.8
HLA-A*24:02	1	937	945	9	NFGAISSVL	NFGAISSVL	NFGAISSVL	0.266724	0.83
HLA-A*24:02	1	360	368	9	YNSTSFSTF	YNSTSFSTF	YNSTSFSTF	0.265817	0.83
HLA-A*24:02	1	1035	1043	9	YVPSQERNF	YVPSQERNF	YVPSQERNF	0.265797	0.83

HLA-A*24:02	1	370	378	9	CYGVSPSKL	CYGVSPSKL	CYGVSPSKL	0.254642	0.86
HLA-A*24:02	1	258	266	9	VGNLKYTTF	VGNLKYTTF	VGNLKYTTF	0.253426	0.86
HLA-A*24:02	1	749	757	9	VFAQVKQMY	VFAQVKQMY	VFAQVKQMY	0.252841	0.87
HLA-A*24:02	1	476	484	9	EYQATRVVV	EYQATRVVV	EYQATRVVV	0.25192	0.87
HLA-A*24:02	1	1187	1195	9	GFIAGLIAI	GFIAGLIAI	GFIAGLIAI	0.250393	0.87
HLA-A*24:02	1	51	59	9	VLHLTQDYF	VLHLTQDYF	VLHLTQDYF	0.250391	0.87
HLA-A*24:02	1	2	10	9	KVLIFALLF	KVLIFALLF	KVLIFALLF	0.241156	0.9
HLA-A*24:02	1	196	204	9	GFLRVYQTY	GFLRVYQTY	GFLRVYQTY	0.228422	0.95
HLA-A*24:02	1	536	544	9	QFGRDTSDF	QFGRDTSDF	QFGRDTSDF	0.227025	0.96
HLA-A*24:02	1	678	686	9	NSIAIPTNF	NSIAIPTNF	NSIAIPTNF	0.226125	0.96
HLA-A*24:02	1	871	879	9	AYRFNGIGV	AYRFNGIGV	AYRFNGIGV	0.223647	0.97
HLA-A*24:02	1	357	365	9	TVLYNSTSF	TVLYNSTSF	TVLYNSTSF	0.219014	0.98
HLA-A*24:02	1	1108	1116	9	PLQPELDSF	PLQPELDSF	PLQPELDSF	0.215721	0.99
HLA-A*24:02	1	783	791	9	RSFIEDLLF	RSFIEDLLF	RSFIEDLLF	0.199604	1.1
HLA-A*24:02	1	419	427	9	DFTGCVIAW	DFTGCVIAW	DFTGCVIAW	0.187286	1.2
HLA-A*24:02	1	1177	1185	9	YIKWPWYVW	YIKWPWYVW	YIKWPWYVW	0.187168	1.2

Table S7 Top 50 CTL epitopes predicted by IEDB for the strain SARS-CoV-2.

allele	seq_num	start	end	length	peptide	core	icore	score	rank
HLA-A*24:02	1	635	643	9	VYSTGSNVF	VYSTGSNVF	VYSTGSNVF	0.969649	0.01
HLA-A*24:02	1	1208	1216	9	QYIKWPWYI	QYIKWPWYI	QYIKWPWYI	0.96134	0.02
HLA-A*24:02	1	489	497	9	YFPLQSYGF	YFPLQSYGF	YFPLQSYGF	0.94419	0.03
HLA-A*24:02	1	448	456	9	NYNYLYRLF	NYNYLYRLF	NYNYLYRLF	0.935258	0.03
HLA-A*24:02	1	57	65	9	PFFSNVTWF	PFFSNVTWF	PFFSNVTWF	0.872845	0.07
HLA-A*24:02	1	1094	1102	9	VFVSNGTHW	VFVSNGTHW	VFVSNGTHW	0.865816	0.08
HLA-A*24:02	1	78	86	9	RFDNPVLPF	RFDNPVLPF	RFDNPVLPF	0.853763	0.08
HLA-A*24:02	1	169	177	9	EYVSQPFLM	EYVSQPFLM	EYVSQPFLM	0.848368	0.09
HLA-A*24:02	1	144	152	9	YYHKNNKSW	YYHKNNKSW	YYHKNNKSW	0.841566	0.09
HLA-A*24:02	1	507	515	9	PYRVVVLSF	PYRVVVLSF	PYRVVVLSF	0.816608	0.1
HLA-A*24:02	1	1137	1145	9	VYDPLQPEL	VYDPLQPEL	VYDPLQPEL	0.81018	0.11

HLA-A*24:02	1	312	320	9	IYQTSNFRV	IYQTSNFRV	IYQTSNFRV	0.784957	0.12
HLA-A*24:02	1	193	201	9	VFKNIDGYF	VFKNIDGYF	VFKNIDGYF	0.756375	0.14
HLA-A*24:02	1	1101	1109	9	HWFVTQRNF	HWFVTQRNF	HWFVTQRNF	0.75401	0.14
HLA-A*24:02	1	706	714	9	AYSNNSIAI	AYSNNSIAI	AYSNNSIAI	0.711972	0.17
HLA-A*24:02	1	268	276	9	GYLQPRTFL	GYLQPRTFL	GYLQPRTFL	0.699837	0.18
HLA-A*24:02	1	504	512	9	GYQPYRVVV	GYQPYRVVV	GYQPYRVVV	0.572097	0.31
HLA-A*24:02	1	755	763	9	QYGSFCTQL	QYGSFCTQL	QYGSFCTQL	0.561401	0.31
HLA-A*24:02	1	350	358	9	VYAWNRKRI	VYAWNRKRI	VYAWNRKRI	0.545957	0.33
HLA-A*24:02	1	167	175	9	TFEYVSQPF	TFEYVSQPF	TFEYVSQPF	0.536479	0.35
HLA-A*24:02	1	269	277	9	YLQPRTFLL	YLQPRTFLL	YLQPRTFLL	0.535929	0.35
HLA-A*24:02	1	1216	1224	9	IWLGFIAGL	IWLGFIAGL	IWLGFIAGL	0.516336	0.37
HLA-A*24:02	1	151	159	9	SWMESEFRV	SWMESEFRV	SWMESEFRV	0.504649	0.39
HLA-A*24:02	1	880	888	9	GTITSGWTF	GTITSGWTF	GTITSGWTF	0.421581	0.49
HLA-A*24:02	1	47	55	9	VLHSTQDLF	VLHSTQDLF	VLHSTQDLF	0.403828	0.52
HLA-A*24:02	1	379	387	9	CYGVSPTKL	CYGVSPTKL	CYGVSPTKL	0.379021	0.55
HLA-A*24:02	1	898	906	9	FAMQMAYRF	FAMQMAYRF	FAMQMAYRF	0.375038	0.56
HLA-A*24:02	1	1095	1103	9	FVSNGTHWF	FVSNGTHWF	FVSNGTHWF	0.330624	0.66
HLA-A*24:02	1	369	377	9	YNSASFSTF	YNSASFSTF	YNSASFSTF	0.32233	0.67
HLA-A*24:02	1	712	720	9	IAIPTNFTI	IAIPTNFTI	IAIPTNFTI	0.285645	0.78
HLA-A*24:02	1	267	275	9	VGYLQPRTF	VGYLQPRTF	VGYLQPRTF	0.273664	0.81
HLA-A*24:02	1	969	977	9	NFGAISSVL	NFGAISSVL	NFGAISSVL	0.266724	0.83
HLA-A*24:02	1	50	58	9	STQDLFLPF	STQDLFLPF	STQDLFLPF	0.264807	0.83
HLA-A*24:02	1	1219	1227	9	GFIAGLIAI	GFIAGLIAI	GFIAGLIAI	0.250393	0.87
HLA-A*24:02	1	781	789	9	VFAQVKQIY	VFAQVKQIY	VFAQVKQIY	0.234418	0.92
HLA-A*24:02	1	366	374	9	SVLYNSASF	SVLYNSASF	SVLYNSASF	0.233864	0.92
HLA-A*24:02	1	710	718	9	NSIAIPTNF	NSIAIPTNF	NSIAIPTNF	0.226125	0.96
HLA-A*24:02	1	903	911	9	AYRFNGIGV	AYRFNGIGV	AYRFNGIGV	0.223647	0.97
HLA-A*24:02	1	1067	1075	9	YVPAQEKNF	YVPAQEKNF	YVPAQEKNF	0.221693	0.98
HLA-A*24:02	1	1140	1148	9	PLQPELDSF	PLQPELDSF	PLQPELDSF	0.215721	0.99

HLA-A*24:02	1	1088	1096	9	HFPREGVFV	HFPREGVFV	HFPREGVFV	0.21378	1
HLA-A*24:02	1	334	342	9	NLCPFGEVF	NLCPFGEVF	NLCPFGEVF	0.210434	1.1
HLA-A*24:02	1	51	59	9	TQDLFLPFF	TQDLFLPFF	TQDLFLPFF	0.208052	1.1
HLA-A*24:02	1	815	823	9	RSFIEDLLF	RSFIEDLLF	RSFIEDLLF	0.199604	1.1
HLA-A*24:02	1	1054	1062	9	QSAPHGVVF	QSAPHGVVF	QSAPHGVVF	0.196138	1.1
HLA-A*24:02	1	584	592	9	ILDITPCSF	ILDITPCSF	ILDITPCSF	0.19522	1.1
HLA-A*24:02	1	428	436	9	DFTGCVIAW	DFTGCVIAW	DFTGCVIAW	0.187286	1.2
HLA-A*24:02	1	54	62	9	LFLPFFSNV	LFLPFFSNV	LFLPFFSNV	0.17575	1.2
HLA-A*24:02	1	926	934	9	QFNSAIGKI	QFNSAIGKI	QFNSAIGKI	0.174848	1.2
